# Combining MRI radiomics, hypoxia gene signature score and clinical variables for prediction of biochemical recurrence-free survival after radiotherapy in prostate cancer

**DOI:** 10.1007/s11547-025-02037-4

**Published:** 2025-07-02

**Authors:** Jim Zhong, Angela Davey, Russell Frood, Alan McWilliam, Jane Shortall, Mark Reardon, Kimberley Reaves, Martin Swinton, Oliver Hulson, Catharine West, David Buckley, Sarah Brown, Ananya Choudhury, Peter Hoskin, Ann Henry, Andrew Scarsbrook

**Affiliations:** 1 Leeds Institute of Medical Research, University of Leeds, Leeds, UK; 2 Department of Radiology, Leeds Cancer Centre, St James’s University Hospital, Leeds Teaching Hospitals National Health Service (NHS) Trust, Beckett Street, Leeds, LS9 7TF UK; 3 Division of Cancer Sciences, School of Medical Sciences, Faculty of Biology, Medicine and Health, The University of Manchester, Manchester, UK; 4 Department of Radiotherapy Related Research, The Christie NHS Foundation Trust, Manchester, UK; 5 Leeds Cancer Research UK Clinical Trials Unit, Trials Research, Leeds Institute of Clinical, University of Leeds, Leeds, UK; 6 Department of Clinical Oncology, Leeds Cancer Centre, St James’s University Hospital, Leeds Teaching Hospitals NHS Trust, Beckett Street, Leeds, LS9 7TF UK

**Keywords:** Prostate cancer, Magnetic resonance imaging, Radiomics, Hypoxia, Radiotherapy

## Abstract

**Purpose:**

To investigate the value of combining MRI radiomic and hypoxia-associated gene signature information with clinical data for predicting biochemical recurrence-free survival (BCRFS) after radiotherapy for prostate cancer.

**Methods:**

Patients with biopsy-proven prostate cancer, hypoxia-associated gene signature scores and pre-treatment MRI who received radiotherapy between 01/12/2007 and 31/08/2013 at two cancer centres were included in this retrospective cohort analysis. Prostate segmentation was performed on axial T2-weighted sequences using RayStation (v9.1). Histogram standardisation was applied prior to radiomic feature (RF) extraction. PyRadiomics (v3.0.1) was used to extract RFs for analysis. Four multivariable Cox proportional hazards BCRFS prediction models using clinical information alone and in combination with RFs and/or hypoxia scores were evaluated using concordance index (C-index) [confidence intervals (CI)]. Akaike Information Criterion (AIC) was used to assess model fit.

**Results:**

178 patients were included. The clinical-only model performance C-index score was 0.69 [0.64–0.7]. The combined clinical-radiomics model (C-index 0.70[0.66–0.73]) and clinical-radiomics-hypoxia model (C-index 0.70[0.65–0.73]) both had higher model performance. The clinical-hypoxia model (C-index 0.68 [0.63–0.7) had lower model performance. Based on AIC, addition of RFs to clinical variables alone improved model performance (*p* = 0.027), whereas adding hypoxia gene signature scores did not (*p* = 0.625). The selected features of the combined clinical-radiomics model included age, ISUP grade, tumour stage, and wavelet-derived grey level co-occurrence matrix (GLCM) RFs.

**Conclusion:**

Adding pre-treatment prostate MRI-derived radiomic features to a clinical model improves accuracy of predicting BCRFS after prostate radiotherapy, however addition of hypoxia gene signatures does not improve model accuracy.

**Supplementary Information:**

The online version contains supplementary material available at 10.1007/s11547-025-02037-4.

## Introduction

Prostate cancer is the commonest malignancy in men and a major cause of cancer-related death [1]. Radiation therapy (RT), including external beam radiation therapy (EBRT) and brachytherapy (BT), is an effective treatment for localised prostate cancer [2]. Despite advances in diagnostic imaging and RT delivery techniques, 30–50% of men with high-risk disease experience biochemical recurrence (BCR) within 10 years of treatment, most commonly due to intraprostatic relapse [3, 4]. BCR is associated with worse outcomes in prostate cancer in terms of local recurrence, distant metastasis, and death [5]. Predicting the likelihood of progression of prostate cancer in individual patients could help oncologists personalise treatment plans and stratify intensity of follow-up appointments tailored to recurrence risk. This would allow earlier detection of disease progression or recurrence and facilitate timely interventions.

Currently, risk stratification in localised prostate cancer predominantly relies on pathological findings from biopsies and standard imaging evaluation to determine spread of disease. Using information on serum prostate specific antigen (PSA) level, tumour stage (T-stage) and Gleason grade allows stratification into three major groups (low-risk, intermediate-risk and high-risk) based on probability of biochemical recurrence after local therapy [6]. Early efforts to incorporate genomics into risk prediction tools have been promising, with Spratt et al. proposing a system integrating existing genomic and clinical information to improve risk stratification [7]. Their combined clinical–genomic risk system better predicted metastasis than using the standard National Comprehensive Cancer Network (NCCN) risk group alone and reclassified 30% of patients.

Hypoxia, a state of low oxygen, is a common micro-environmental feature in most solid tumours, which activates multiple biological processes such as glycolysis and angiogenesis, inducing the expression (mRNA abundance) of multiple genes involved in these pathways and changes in transcriptomic profiles [8]. High-throughput expression profiling technologies that can measure RNA expression have allowed the development of hypoxia-associated gene signatures, which were prognostic and associated with RT resistance and metastatic disease in prostate cancer cohorts [9–12].

Traditionally, measuring oxygen levels in tumours has been performed using needle electrodes, however this is invasive, technically demanding, and not representative of the whole prostate [13]. Magnetic resonance imaging (MRI) has an essential role in prostate cancer for diagnosis and treatment planning with the potential for monitoring after therapy to assess local recurrence [14]. When combined with radiomics, a quantitative image analysis technique used to derive imaging biomarkers [15], MRI-based radiomic prognostic models have shown improved prediction of survival outcomes for multiple cancer types compared with clinical information alone [16–18]. Additionally, combining MR-imaging, which can detect characteristics (phenotypes) associated with more aggressive prostate disease [19], with gene-based biomarkers shows promise in aiding prediction of clinical outcomes such as survival or treatment resistance [20, 21].

Recent work has demonstrated a potential association between radiomic features (RF) derived from MRI and pimonidazole-based hypoxia biomarkers, showing it may be feasible to develop a radiomics hypoxia model using T2-weighted (T2w) sequences [22]. There is limited evidence on the utility of combining imaging and hypoxia-associated genomic biomarkers for outcome prediction. To the best of our knowledge, only a single study (in cervical cancer) has evaluated integrated imaging and gene expression signatures for non-invasive assessment of hypoxia-related treatment resistance [21]. Identifying imaging “radiogenomic” (combined radiomic and genomic) hypoxia signatures may potentially offer a non-invasive way to analyse the whole prostate and predict outcome.

The study aim was to investigate the value of combining prostate MRI radiomic and hypoxia-associated gene signature information with clinical data for the prediction of biochemical recurrence-free survival (BCRFS) in men with prostate cancer treated with radiotherapy.

## Methods

### Study design

Prostate cancer patients treated with primary radiotherapy between 01/12/2007 and 31/08/2013 at two UK NHS hospitals were included in this retrospective cohort study. The study was approved by the United Kingdom North West Research Ethics Committee (IRAS 15/NW/0559). All patients also received androgen deprivation therapy.

Inclusion criteria were: (a) male patients with organ-confined or locally advanced prostate cancer (with no detected nodal disease or distant metastatic), aged at least 18 years; (b) biopsy-confirmed high-risk prostate cancer; (c) primary radiotherapy to treat their prostate cancer ( EBRT ± BT); (d) available pre-treatment MRI; (e) available formalin-fixed, paraffin-embedded (FFPE) biopsy to enable Ragnum and West hypoxia gene signature evaluation; (f) available clinical features (patient age, International Society of Urological Pathology (ISUP) grade, PSA and T-stage) and clinical outcome data.

Biochemical recurrence (BCR) after radiotherapy was defined by a PSA rise ≥ 2 ng/ml above the nadir.

Diagnostic MR images, patient and tumour characteristics (ISUP grade and T-stage), and hypoxia gene signature were collated for all patients. Adherence was made to the updated 2024 Checklist for Artificial Intelligence in Medical Imaging (CLAIM) (Supplementary Material), a tool for assessing the quality of multivariate prediction models involving imaging and machine learning (ML) techniques [23].

### MRI acquisition

All patients underwent prostate imaging on 1.5 T MRI scanners which included a minimum of an axial T2w sequence encompassing the whole prostate. Imaging was performed using multiple different MRI scanners. Specific scanner acquisition parameters are listed in Supplementary Material.

### Hypoxia-associated gene signatures

Two different hypoxia gene signatures were used: a 32-gene signature based on pimonidazole staining (Ragnum) and a 28-gene signature (West) based on hypoxia induced expression in prostate cancer cell lines. Both signatures were prognostic for biochemical recurrence in several prostatectomy cohorts [11, 12]. For the cohorts studied here, signature scores were generated from gene expression data generated using Affymetrix GeneChip Clariom™ S microarrays after RNA extraction from archived pre-treatment tumour samples. Expression data passing quality control checks were normalised into an expression matrix [24]. The gene enrichment analysis and construction of the gene signatures has also been previously described [12].

### Methodological pipeline

**Fig. 1:**
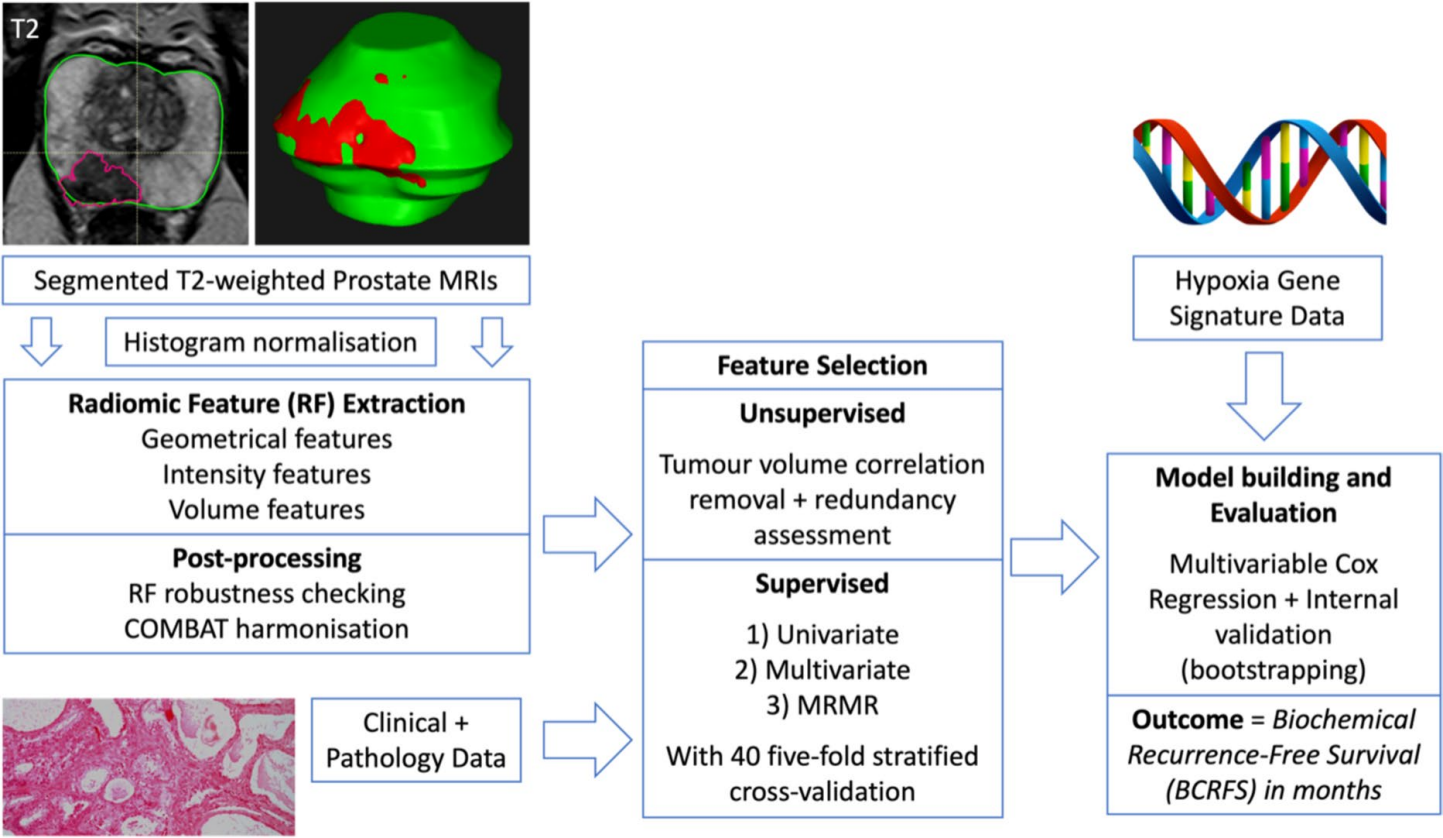
Flowchart showing study pipeline from image segmentation, image normalisation, radiomic feature extraction, image post-processing, feature selection steps to model building integrating hypoxia and radiomic data with clinical data. COMBAT, combating batch effects when combining batches; MRMR, minimum redundancy maximum relevance

A flowchart illustrating the methodological pipeline is shown in Fig. 1.

### Image segmentation & histogram normalisation

The whole prostate gland and prostate tumour (if visible) were manually segmented by an experienced radiologist and confirmed by a specialist Uroradiologist. All segmentation was performed on axial T2w sequences using RayStation (v9.1). Exported DICOM images were converted to Neuroimaging Informatics Technology Initiative (NIfTI) files using Python (v. 3.0.1, dicom2nifti package) and exported into PyRadiomics (v3.0.1) for analysis [25]. Histogram standardisation normalisation of all MR-images was applied using the Nyúl method prior to RF extraction to render dynamic signal intensity ranges comparable [26].

### Radiomic feature extraction

Eight RF classes were extracted from each segmented region of interest (ROI) using PyRadiomics (v3.0.1) (25). PyRadiomics deviates from the Image Biomarker Standardisation Initiative (IBSI) by default applying a fixed bin width from zero and not the minimum segmentation value, and the PyRadiomics kurtosis is not corrected, yielding a value three higher than the IBSI kurtosis however these parameters can be manually adjusted. Otherwise, PyRadiomics adheres to IBSI guidelines, which provide a comprehensive review of each step involved in radiomic analyses, including radiomics nomenclature and required calibration datasets [27]. All RFs extracted and filters applied are detailed in Supplementary Material Table 2.

### Post-processing

Different numbers of bins (8, 16, 32, 64, 128, 256) and isotropic voxel sizes (1, 2, 3) were tested to assess the most robust quantisation/re-binning setting based on the combination of bin number and voxel size that yielded the largest set of radiomic features. An intraclass correlation coefficient (ICC) threshold of > 0.8 was also used to eliminate inter-correlated features. Bin number was favoured over the bin width given the arbitrary nature of MRI intensity units. COMBAT harmonisation (v0.2.10) was applied to extracted RFs to account for variation in scanner models, acquisition protocols and reconstruction settings which RFs are affected by [28, 29].

### Feature selection

RF feature selection was focused on ability to predict biochemical recurrence.

- Unsupervised feature selection
  For each RF, correlation with tumour volume was assessed with Spearman rank correlation coefficient (p) and features with a p-value > 0.5 were removed. RFs were assessed for redundancy (linear correlation to other RFs) using Pearson correlation coefficient. If the correlation coefficient was 0.5 or higher between two RFs then they were deemed to be correlated and the feature in the correlated pair with the highest mean correlation to other RFs was removed.
- Supervised feature selection
  Following a previously published approach [30], supervised feature selection was performed using three different techniques for comparison. The methods implemented selected features that: (1) were significantly associated with outcome (i.e. BCRFS) in a univariable Cox regression model (*p* < 0.05), (2) significantly improved a multivariable Cox regression model of clinical variables in a likelihood-ratio (LR) test (*p* < 0.05), and (3) had a positive contribution based on minimum redundancy maximum relevance (MRMR) ranking [31].

Each feature selection method was implemented independently over 200 samples created from 40 five-fold stratified cross-validation (SCV) runs with event-matching for number of biochemical recurrences, meaning the data was partitioned into five sub-sets, four for training and one held for testing with the number of events balanced between the subsets. Training was repeated five times with each subset being held as the test set independently. The separation of the subsets was repeated 40 times, with overall 200 cases to test model performance on. In each cross-validation training run, selected features were combined with clinical variables (age, ISUP grade, PSA, T-stage, tumour volume and treatment) to form a clinical-radiomics multivariable Cox model. This model was then applied to the test data. Including the clinical variables for the other feature selection methods (univariable, MRMR) made the comparison to the multivariable feature selection technique fair. Harrell’s concordance index (C-index) was calculated for both training and test models with the median and 95% CI across SCV runs recorded. The feature selection technique was selected based on calculating performance ranking from the median C-index across all clinical-radiomic models for both training (Ctrain) and test (Ctest) data [32]. For the chosen technique, the selected features from each training run were recorded and ranked by occurrence. The top ranking features up to the median number of features selected across all runs were recorded.

### Model building

Using the feature results from the chosen feature selection technique, four different multivariable Cox proportional hazards models were constructed for comparison: 1) clinical only, 2) clinical + hypoxia, 3) clinical + radiomics, and 4) clinical + hypoxia + radiomics to demonstrate whether adding hypoxia and radiomic data improved overall model performance compared to clinical information alone, the gold standard used as per the radiomics quality score guidance [15]. The Akaike Information Criterion (AIC) was extracted, and an Analysis of Variance (ANOVA) test was used to compare if there was a significant difference in regression model performance for each model 2) to 5), in comparison to model 1) (clinical only model as baseline). AIC provides a mathematical method to evaluate how well a model fits the data it was generated from [33]. A smaller AIC value indicated a better goodness of fit for predicting outcomes.

For internal testing, the median and 95% CIs of the C-index for each model across 500 bootstrap resamples was calculated. C-index was calculated and each of these bootstrap models was fitted to the original data. For analysis, T-stage was grouped into T1/2 and T3 groups. Radiomic features were scaled to have a mean of zero, and a unit variance of one.

Statistical analysis was performed in R (v.4.0.2). Two-tailed tests were used with statistical significance defined as *p* < 0.05.

## Results

### Clinical characteristics

A total of 178 patients with histologically confirmed prostate cancer were treated with either EBRT (74 Gy in 37 fractions) (n = 143), or EBRT (37.5 Gy in 15 fractions) plus high dose rate (HDR) brachytherapy (BT) boost (single fraction 15 Gy) (n = 35), between 01/12/2007 and 31/08/2013. All patients received androgen deprivation therapy (ADT).

The clinical and treatment characteristics for all patients are listed in Table 1. Complete clinical, hypoxia and radiomics data was available for all patients. Median follow-up was 84 months (range 3–140). BCR rate was 32% (n = 60). Median BCRFS was 74 months (range 2–132).

**Table 1:** Demographics of the study cohort

Characteristic	N = 178^1^
Age (years)	70 (52–80)
PSA (ng/mL)	20 (2–234)
*ISUP*	
1	5 (2.8%)
2	66 (37%)
3	35 (20%)
4	16 (9.0%)
5	56 (31%)
*T-stage*	
T1	4 (2.2%)
T2	34 (19%)
T3	139 (78%)
T4	1 (0.6%)
Tumour volume (ml)	5 (0–97)
*Treatment*	
EBRT	143 (80%)
HDR + EBRT	35 (20%)
Ragnum-32 hypoxia score	0.30 (− 0.37–0.82)
West-28 hypoxia score	1.40 (− 2.05–3.65)

PSA, prostate specific antigen; ISUP, international society of urological pathology; EBRT, external beam radiotherapy; HDR-BT, high dose rate brachytherapy

^1^Statistics presented: n (%); Median (range)

### Radiomic feature selection

The combination of bin number 256 and voxel size one yielded the greatest number of robust radiomic features. A total of 1314 RFs were extracted, 1068 remained after volume correlation, and 55 remained after removals for redundancy. The median number of RFs selected was seven by MRMR, three in multivariable, and three in univariable. The univariable technique had the best model performance across training and test data with a test C-index of 0.61 compared to multivariable (C-index 0.60) and MRMR model (C-index 0.59).

**Fig. 2:**
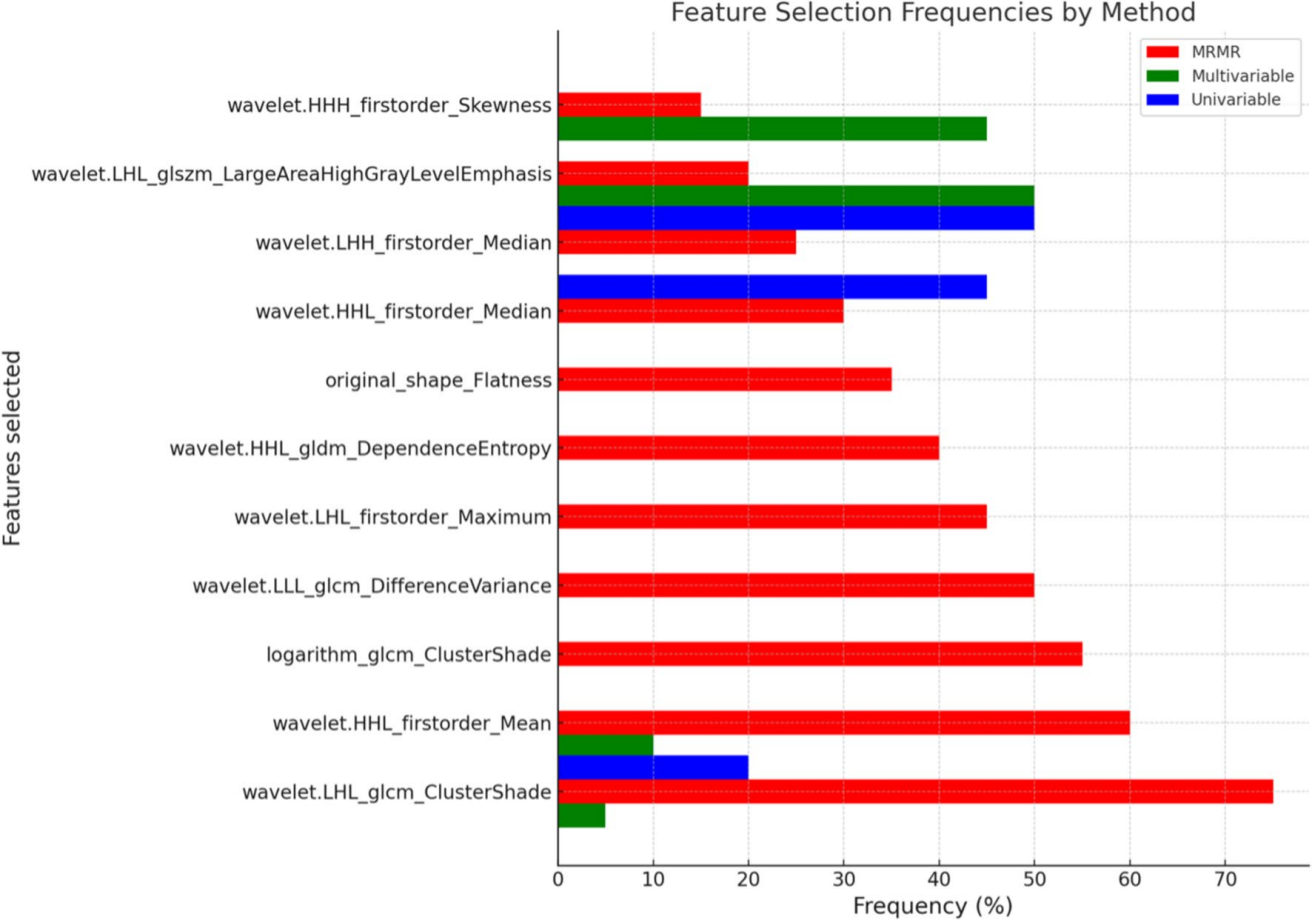
Bar chart showing the frequency (%) that each radiomic feature was selected across all cross-validation runs (out of 200) for each feature selection method. LHH, HHL, HLH, LLL, 3D wavelet radiomic features; GLRLM, grey level run length matrix; GLCM, grey level co-occurrence matrix; GLDM, grey level dependence matrix; GLSZM, grey level size zone matrix

Figure 2 shows the frequency (%) that each feature was selected across all cross-validation runs (out of 200). The higher the frequency the more stable that feature is in the feature selection process.

### Prediction model performance

**Fig. 3:**
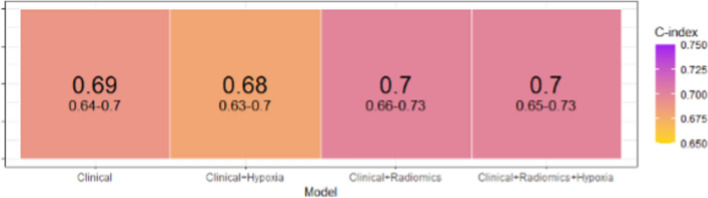
C-index and confidence interval (CI) of all 4 models, showing the joint best models were Clinical + Radiomics (0.7) and Clinical + Radiomics + Hypoxia (0.7)

When evaluated on the complete dataset, the median C-index and confidence intervals (CI) of all four prediction models are shown in Fig. 3.

Based on the C-index, the combined clinical-radiomics model (C-index 0.7[0.66–0.73]) and clinical-radiomics-hypoxia model (C-index 0.70[0.65–0.73]) had the joint highest model performance. The clinical-only model (C-index of 0.69 [0.64–0.7]) and clinical-hypoxia model (C-index of 0.68 [0.63–0.7) had lower model performance.

Each model and overall model fit based on AIC are presented in Table 2.

**Table 2:** Selected features for each of the four models (clinical only, clinical with hypoxia, clinical with radiomics and combined clinical, hypoxia and radiomics) and overall model performance score (AIC Statistic)

	Clinical model		Clinical + Hypoxia		Clinical + Radiomics		Clinical + Radiomics + Hypoxia	
	HR (95% CI)	*P*-value	HR (95% CI)	*P*-value	HR (95% CI)	*P*-value	HR (95% CI)	*P*-value
Age (years)	0.95 (0.91–0.99)	**0.012**	0.95 (0.91–0.99)	**0.01**	0.96 (0.92–1)	**0.037**	0.95 (0.91–1)	**0.029**
PSA (ng/mL)	1.01 (1–1.01)	0.061	1.01 (1–1.01)	0.07	1.01 (1–1.01)	0.069	1.01 (1–1.01)	0.075
ISUP grade	1.33 (1.07–1.64)	**0.008**	1.32 (1.07–1.63)	**0.01**	1.32 (1.06–1.63)	**0.011**	1.3 (1.05–1.62)	**0.017**
T-stage (T1/2 vs T3)	2.88 (1.18–7.06)	**0.021**	2.9 (1.18–7.1)	**0.02**	3.24 (1.29–8.15)	**0.013**	3.2 (1.27–8.1)	**0.014**
Tumour volume (ml)	1 (0.98–1.03)	0.682	1.01 (0.98–1.03)	0.645	1 (0.98–1.03)	0.733	1 (0.98–1.03)	0.693
Treatment (EBRT vs HDR-BT)	1.55 (0.77–3.1)	0.22	1.51 (0.75–3.03)	0.247	1.31 (0.63–2.74)	0.466	1.29 (0.62–2.69)	0.491
Ragnum-32 hypoxia score	NA	NA	1.37 (0.37–5.04)	0.637	NA	NA	1.45 (0.37–5.69)	0.597
West-28 hypoxia score	NA	NA	0.93 (0.7–1.23)	0.599	NA	NA	0.93 (0.7–1.25)	0.644
Wavelet HHL first order median	NA	NA	NA	NA	0.78 (0.56–1.09)	0.145	0.79 (0.56–1.11)	0.167
Wavelet LHL GLCM cluster shade	NA	NA	NA	NA	1.38 (1.08–1.76)	**0.01**	1.37 (1.07–1.75)	**0.012**
Wavelet LHH first order median	NA	NA	NA	NA	0.81 (0.6–1.09)	0.173	0.8 (0.59–1.08)	0.141
AIC statistic	*481.86*		*485.34*		*478.71*		*482.22*	
*Comparison to clinical model			*P = *0.774*		*P = *0.027*		*P = *0.086*	

The radiomic features are scaled to mean zero and unit variance

The significant variables (*p* < 0.05) are highlighted in bold

EBRT, external beam radiotherapy; HDR-BT, high dose-rate brachytherapy; HHL, HLH, LHH, LLL, 3D wavelet radiomic features; GLCM, grey level co-occurrence matrix; AIC, akaike information criterion

The combined clinical and radiomics model has the lowest AIC (AIC = 478.71) and best model fit. When comparing the combined model AICs to the clinical only model using an ANOVA test, the clinical and radiomics model was statistically significantly better (*p* = 0.027, (*p* = exp(-ΔAIC/2)[34]). Including hypoxia information alone (clinical + hypoxia model) did not improve model performance (*p* = 0.774).

## Discussion

This study demonstrates the feasibility of combining pre-treatment T2w MRI-derived radiomic features with standard clinical variables to help improve performance of predicting BCRFS after prostate radiotherapy and ADT. While hypoxia-associated gene signatures have been shown to be prognostic in men treated primarily with surgery, they did not improve the model performance further in this bi-institutional cohort. A strength of this study is the unique dataset of paired imaging and genomic data available from two centres.

The results of this study are supported by existing literature where the utility of MRI-based radiomic analysis in prostate cancer has been shown to be feasible in cancer diagnosis and the prediction of Gleason score [35–38]. More recently, prediction models using prostate MRI radiomics have been reported assessing risk of BCR after radiotherapy. Gnep et al. demonstrated that T2w MRI-derived Haralick textural features, which quantify spatial relationships between neighbouring voxels, were associated with BCR occurrence [39] (48). Few studies have investigated the role of MRI-derived radiomics in assessing progression-free survival in prostate cancer, however an initial report of 191 patients combining radiomics and clinical data into a hybrid prediction model yielded excellent performance with AUCs of 0.926 and 0.917 in the training and internal testing groups, respectively, which shows promise as a non-invasive diagnostic tool for risk stratification [16]. Two RFs selected across all the feature selection methods used in this study were GLSZM Large Area High Gray Level Emphasis and First Order Mean, which quantifies grey level zones (the number of connected voxels that share the same grey level intensity) in an image [25]. A recent study of 63 men receiving carbon ion radiotherapy for prostate cancer found similar RFs relating to Grey Level Zones were able to both predict the tumour metabolomics, such as alteration in the methionine amplitude and BCR. The current study also supports the notion that whole prostate gland radiomic features can provide additional information to help predict BCR.

Imaging and genomic biomarkers have different strengths, there are a paucity of studies investigating how they relate to each other in prostate cancer and their impact on survival outcomes to understand how to fully exploit any synergistic potential, the rationale for the current study. In cervical cancer, a multimodal prediction model combining both imaging and gene expression signatures in 118 patients was studied to assess hypoxia-related treatment resistance, a combined model allowed better prediction of progression-free survival[21]. In our prostate cancer study the addition of two prostate specific hypoxia-associated gene signatures did not improve prediction of BCRFS however the proviso is that the current sample remains small, and hypoxia signatures were not prognostic despite other previous studies showing the signatures being prognostic for BCRFS in several external patient cohorts, albeit most being treated with prostatectomy [11, 12]. Imaging permits more holistic prostate assessment compared to a biopsy-derived hypoxia gene signature limited to the sampled region/s which may not capture the overall spatial and temporal tumour heterogeneity [40]. One limitation of imaging however is the difficulty in identifying the dominant intraprostatic lesion (DIL), particularly after ADT, which would make delineating and extracting the radiomics from the DIL challenging [41]. Regional differences in hypoxia exist across the entire tumour volume, and this heterogeneity may limit the utility of gene signatures derived from limited parts of the tumour [42]. This may help explain why the addition of the hypoxia-associated gene signature did not improve performance of outcome prediction in this study. However, recent work testing the signatures in radiotherapy cohorts reported a lack of prognostic significance in men treated with radiotherapy and ADT, and suggested that use of hormone therapy might have reduced the effect of hypoxia [43]. Therefore, the lack of benefit of adding hypoxia signature might also be due to their poor performance in men receiving ADT with radiotherapy, which is highly relevant to this study cohort.

The tumour ISUP grade, derived from biopsy, was prognostic of BCR as previously highlighted in the literature [44]. ISUP grades are categorical and a study by De Nunzio et al. found the rate of discrepancy between biopsy pathology and the prostatectomy specimen to be low meaning the five tier ISUP system was highly specific (91%) for correctly defining the tumour aggressiveness however hypoxia gene signature scores are more prone to fluctuations caused by sample preparation and potential RNA degradation due to longer storage times [45, 46]. Based on this knowledge, studying two different hypoxia signatures was important in understanding the relationship and interaction between hypoxia scores and other clinical, biochemical and pathological markers in a multivariate prediction model.

Imaging features could be linked to underlying biological changes and the potentially predictive prostate RFs observed in this study might be a surrogate for tumour aggressiveness and hypoxia. Wavelet transformation of RFs further separates out the spatial and frequency distributions of low and high frequency signals within the region of interest to delineate such changes [47]. Differentiating these properties may improve the overall performance of the hybrid radiomic prediction model, as demonstrated by the current study where the best performing radiomic features were all wavelet ones. Recent work has reported associations between T2w MRI radiomic features of the whole prostate gland or index lesion and tumour hypoxia, demonstrating the feasibility of building a radiomics hypoxia model from anatomical MRI [22, 48]. A study of 15 patients found a correlation between bi-parametric prostate MRI radiomic features extracted in localised prostate cancer and differentially expressed hypoxia-related genes associated with unfavourable survival outcomes [49]. The potential link between the imaging and gene signatures requires further investigation in prostate cancer to find surrogate measures that could be used in prognosticating patients.

There are a number of limitations to the study. Our study was retrospective with MRI data acquired from several scanners across different institutions, which is why an image harmonisation method was applied to minimise bias. There is also a limitation in using gene signatures because they require expression profiling platforms to measure the relative mRNA abundance, which is affected by the biopsy sample preservation technique (e.g. fresh-frozen or FFPE), age of the FFPE blocks and by technical batch effects which may limit the reliability of generating hypoxia scores between different institution cohorts. Both signatures were derived using gene expression data generated from fresh frozen samples but were obtained using FFPE material in our study. Other limitations in generating hypoxia scores were minimised by carrying out RNA extractions and gene expression profiling at a single centre so the same methods and platforms were used on the cohorts. The use of two hypoxia signatures was also a strength of this study as they were generated using different approaches. The Ragnum signature was trained on pimondiazole staining and the West signature was derived by identifying genes first in vitro via RNA-sequencing of prostate cancer cell lines and then in vivo via gene co-expression analysis.

Finally, the choice of outcome metric remains debateable as BCRFS was not a surrogate endpoint for overall survival in recurrent prostate cancer in the NRG Oncology/RTOG 9601 phase III trial [50]. BCR may be due to local or systemic relapse, both of which hypoxia predisposes to and hypoxia-associated gene signatures have been identified as independent risk factors for metastasis-free survival in prostate cancer [51], therefore evaluating the prediction of other survival endpoints may be more widely accepted by the clinical oncology community. The challenge of using overall survival is however the loss of follow-up for many of the included patients due to them being followed up only within the primary care setting which was not traceable.

Only T2w imaging was used due to the historic nature of MRIs available in participants with matched genomic data. Studies in cervical cancer have found that a radiomic signature derived from diffusion-weighted imaging (DWI), a functional sequence with quantitative information reflecting cellularity, outperformed a model using T2w MRI-derived RFs for predicting survival [17]. In prostate cancer, a combined DWI and T2w survival prediction model outperformed models using only one of these sequences when predicting 3-year progression-free survival [16]. It is reasonable to assume DWI and apparent diffusion coefficient (ADC) information would add to the prognostic information offered by the T2w sequence, which is mainly used for detailing anatomy, whereas DWI/ADC measure underlying tumour cell density and water diffusion which can provide additional information on the cellular microenvironment and even hypoxia [42]. A previous study mapping whole-mount prostate pathology specimens with MRI images was able to use machine learning to generate predictive maps of pathologic features and regions of high-grade tumours based on MRI alone, with ADC features being the most highly predictive [52]. This has direct implications for the radiation oncology. In theory, using a radiogenomics or hypoxia-driven biological adapted radiotherapy treatment approach could facilitate more accurate delivery of focal radiation boosts to the ‘hypoxic’ or most aggressive parts of a tumour, in order to improve oncological outcomes and avoid rectal and urinary bladder toxicity in men treated with hypo-fractionated external beam radiotherapy for localised cancers [53]. To strive towards this future, rigorous additional investigation is first required to see if links between genomic signatures and imaging-based hypoxia signatures can be found. The role of hypoxia functional MRI sequences to generate radiology hypoxia maps and enable detection of hypoxic regions will be a good starting point as preliminary results show the correlation between hypoxia regions on imaging with pimonidazole-stained pathological specimens [42]. Future work will integrate other functional sequences such as DWI/ADC and dynamic contrast-enhanced (DCE) imaging to the prediction model in establish if this improves performance.

## Conclusion

Prediction of BCRFS after prostate radiotherapy using pre-treatment prostate MRI-derived radiomic features is technically feasible and improved model performance when combined with clinical variables. Addition of hypoxia gene signature score did not improve predictive accuracy. Further multicentre testing to assess reproducibility of radiomics is required prior to clinical translation.

## Supplementary Information

Below is the link to the electronic supplementary material.

Supplementary file1 (DOCX 21 KB)
